# Overcoming the Barriers That Obscure the Interlinking and Analysis of Clinical Data Through Harmonization and Incremental Learning

**DOI:** 10.1109/OJEMB.2020.2981258

**Published:** 2020-03-16

**Authors:** Vasileios C. Pezoulas, Konstantina D. Kourou, Fanis Kalatzis, Themis P. Exarchos, Evi Zampeli, Saviana Gandolfo, Andreas Goules, Chiara Baldini, Fotini Skopouli, Salvatore De Vita, Athanasios G. Tzioufas, Dimitrios I. Fotiadis

**Affiliations:** Unit of Medical Technology and Intelligent Information Systems, Department of Materials Science and EngineeringUniversity of Ioannina 37796 GR45110 Ioannina Greece; Unit of Medical Technology and Intelligent Information Systems, Department of Materials Science and EngineeringUniversity of Ioannina 37796 GR45110 Ioannina Greece; Department of Biological Applications and TechnologyUniversity of Ioannina 37796 GR45110 Ioannina Greece; Department of InformaticsIonian University 68997 GR49100 Corfu Greece; Unit of Medical Technology and Intelligent Information Systems, Department of Materials Science and EngineeringUniversity of Ioannina 37796 GR45100 Ioannina Greece; Institute for Systemic Autoimmune and Neurological Diseases GR11743 Athens Greece; Clinic of Rheumatology, Department of Medical and Biological SciencesUdine University IT33100 Udine Italy; Department of Pathophysiology, School of MedicineUniversity of Athens 68993 GR15772 Athens Greece; Department of Clinical and Experimental MedicineUniversity of Pisa 9310 Pisa IT56126 Italy; Department of Internal Medicine and Clinical ImmunologyEuroclinic Hospital GR11521 Athens Greece; Unit of Medical Technology and Intelligent Information Systems, Department of Materials Science and EngineeringUniversity of Ioannina 37796 GR45110 Ioannina Greece; Department of Biomedical ResearchFORTH-IMBB GR45110 Ioannina Greece

**Keywords:** Data sharing, data curation, data harmonization, federated data analytics, incremental learning

## Abstract

*Goal:* To present a framework for data sharing, curation, harmonization and federated data analytics to solve open issues in healthcare, such as, the development of robust disease prediction models. *Methods:* Data curation is applied to remove data inconsistencies. Lexical and semantic matching methods are used to align the structure of the heterogeneous, curated cohort data along with incremental learning algorithms including class imbalance handling and hyperparameter optimization to enable the development of disease prediction models. *Results:* The applicability of the framework is demonstrated in a case study of primary Sjögren's Syndrome, yielding harmonized data with increased quality and more than 85% agreement, along with lymphoma prediction models with more than 80% sensitivity and specificity. *Conclusions:* The framework provides data quality, harmonization and analytics workflows that can enhance the statistical power of heterogeneous clinical data and enables the development of robust models for disease prediction.

## I. Introduction

Nowadays, there are several significant and challenging open issues in healthcare. Examples of such open issues include the sharing and interlinking of clinical data from different clinical databases [1], [2], the enhancement of the quality of the clinical data [3] and the subsequent harmonization of the structurally heterogeneous clinical data [1] in order to increase the overall population size and thus enhance the statistical power of the clinical studies. Once the data are shared, qualified and harmonized, data analytics can be applied to address clinical unmet needs, such as, the development of robust machine learning models for effective disease management, the detection of biomarkers and cost-effective treatments [1], [4]–[6].

Everything starts from data sharing. The data sharing process must take into account legal and ethical issues which are posed during sharing sensitive personal data with the platform according to the existing data protection regulations, such as, the General Data Protection Regulation (GDPR) in Europe [7] and the Health Insurance Portability and Accountability Act (HIPAA) [8] in USA. Once the legal and ethical compliance of the data is ensured, the quality assessment of the clinical data comes next. Indeed, the absence of data curation yields clinical data with poor quality that may lead to erroneous results [9]–[11]. In general, the quality of the clinical data can be enhanced in terms of accuracy, completeness and conformity through a computational process which is known as medical data curation [12]. Once the quality of the clinical data is enhanced, the co-analysis of the curated clinical data comes next. In order to do so, the clinical data must be homogenized through a process which is known as data harmonization [2].

Medical data harmonization overcomes the structural heterogeneities through the identification of lexically or conceptually similar terminologies between two or more heterogeneous clinical datasets. This is most commonly achieved by lexically and/or semantically matching the terms of the heterogeneous datasets using a reference model which serves as a gold standard [1], [13]. The reference model is defined as a set of terminologies which describe the domain knowledge of a disease of interest and is usually expressed in the form of an ontology using classes, sub-classes and object properties describing the relationship between the terms [14]. This set of terms is usually defined by the clinical experts in the field and includes various clinical parameters which are related to laboratory tests, biopsies, treatments, etc. The terminology matching process, however, is not always enough since the values of the matched terminologies need to be transformed according to the pre-defined range values in the reference model. This is done through a process which is known as data standardization and is part of data harmonization [15]. Several data harmonization frameworks have been proposed to co-analyze heterogeneous medical data, including the DataSHaPER [16] framework, the SORTA tool [17], the BiobankConnect software tool [18], the S-match semantic matching framework [19], and finally the FOAM [20]. The majority of these frameworks, however, is mainly focused on the harmonization of genomic data.

Once the data are harmonized they need to be co-analyzed. The conventional approach is to integrate the harmonized data under a common database and apply machine learning to deal with the unmet needs in various medical domains, such as, the development of disease prediction models. A centralized database, however, is prone to privacy breach and computationally inefficient in the case of big data, where the memory and processing requirements are demanding [21], [22]. Towards this direction, batch processing methods have been proposed to deal with the analysis of big data by sequentially fetching the data into smaller subsets, where the machine learning algorithms are applied on an initial batch and then updated on the upcoming batches until all the batches are being processed. Examples of batch processing methods include online learning and incremental learning [23]–[25]. The main difference between them is the fact that the latter does not expect the data to arrive “online” in the form of data streams. Conventional implementations include the SVM with linear kernel [26] stochastic gradient boosting based on ensemble classifiers [27], Naïve Bayes [28], etc.

In this work, we present a complete framework for medical data sharing, curation, harmonization and federated data analytics. The legal and ethical compliance of the data sharing process is ensured through a data sharing assessment mechanism. A medical data curation workflow is applied on the clinical data to deal with outliers, inconsistent fields and missing values. The curated data are then harmonized using lexical and semantic matching methods based on a disease-oriented ontology. The harmonized data are finally stored in private cloud spaces and co-analyzed using incremental learning algorithms to address a clinical unmet need related to lymphoma prediction in primary Sjögren's Syndrome (pSS) using clinical data from four European cohorts. The outcomes of the case study yield highly qualified and harmonized data with more than 85% agreement along with robust lymphoma prediction models with more than 80% accuracy, sensitivity and specificity.

## II. Results

### A. Cohort Data Sharing

We acquired anonymized clinical data from four European cohorts on primary Sjögren's Syndrome (University of Athens (UoA); Harokopion University (HUA); University of Pisa (UNIPI); University of Udine (AOUD)). The cohort data (Supplementary Table I) were shared with the platform under the data protection agreement version 3.7 as of August 2018 according to the Article 35 (3) (b) of the GDPR fulfilling all the necessary ethical and legal requirements for data sharing. In brief, pSS is a chronic inflammatory autoimmune disease causing salivary gland dysfunction, affecting primarily women near the menopausal age [29], [30], where 5% of the pSS patients are associated with the development of non-Hodgkin lymphoma (NHL) [29].

### B. Cohort Data Curation

The cohort data curation workflow (Section V) was applied to enhance the quality of each individual dataset in terms of accuracy, relevance and completeness. All bad features, outliers and inconsistent fields were discarded from further analysis and all fields with missing values were imputed, where applicable. The curated cohort data were finally stored in secure private spaces within the cloud. The extracted cohort metadata are presented in Supplementary Table II. In total, 31 features were inconsistent in the UoA cohort, 6 in the HUA cohort and 1 in the AOUD cohort, where the UoA and HUA cohorts had the highest number of bad features. The total percentage of missing values was 44.8% for the UoA cohort, 33.61% for the HUA cohort, 21.98% for the UNIPI cohort and 17.15% for the AOUD cohort. No outliers were detected across the cohorts.

### C. Cohort Data Harmonization

The cohort data harmonization workflow (Section V) was applied on the curated cohort data. The pSS ontology was used as a gold standard to enable the terminology alignment of each cohort dataset. The number of relevant terms with the pSS reference model was initially identified by the clinical experts. According to Supplementary Table III, the data harmonization process was able to match more than 85% of the reference model terms in all four cohorts (UoA: 92.3%; HUA: 90.47%; UNIPI: 88.88%; AOUD: 89.13%) yielding harmonized data with increased statistical power. Moreover, the number of terms requiring data standardization was significant in the AOUD cohort (14 terms) whereas in the remaining cohorts the terms were already in line with the pre-defined range values in the reference ontology.

**Fig. 1.**
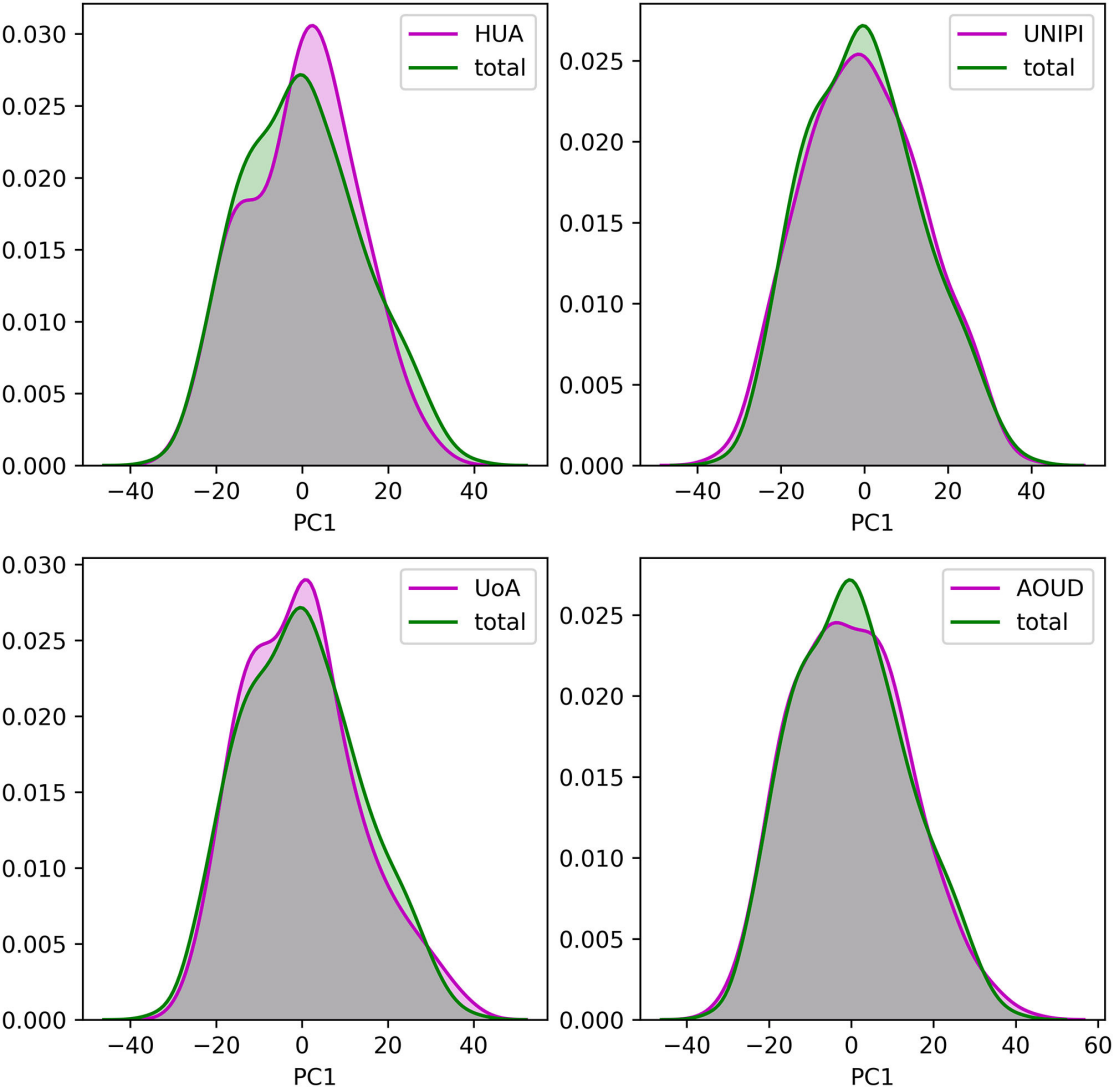
The distribution of the first principal component for each harmonized cohort dataset against the integrated dataset.

**Fig. 2.**
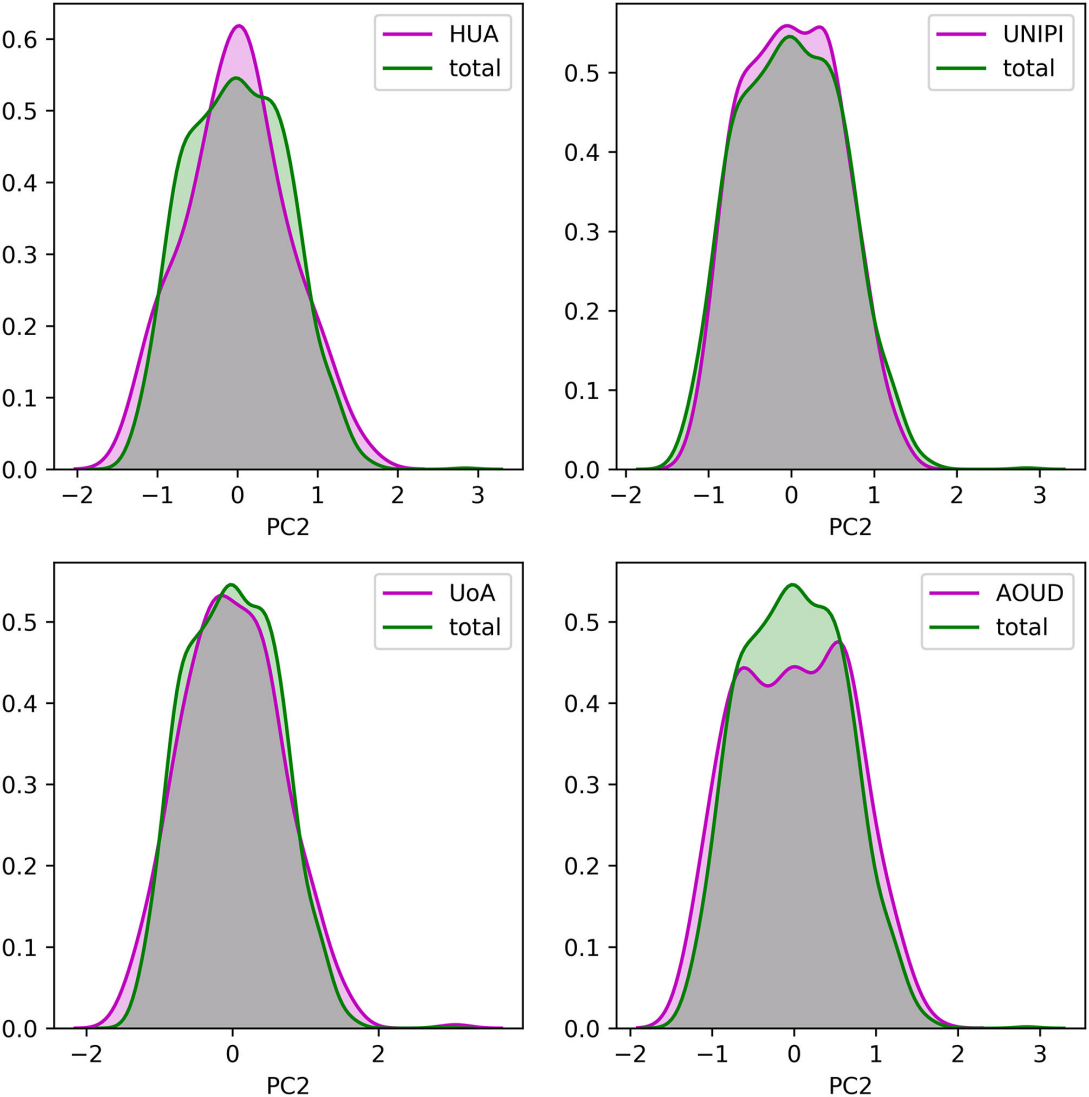
The distribution of the second principal component for each harmonized cohort dataset against the integrated dataset.

To demonstrate the consistency of the harmonized cohort data we applied Principal Component Analysis (PCA) on each harmonized cohort dataset, separately, as well as, on the integrated dataset and extracted the first two principal components (PCs) as those that describe the largest portion of variance within the data. The distributions of the two PCs from each harmonized cohort against those from the integrated cohort, are depicted in Fig. 1 and Fig. 2, respectively. In an attempt to offer a quantitative way to demonstrate the consistency of the data after the data harmonization process, we applied the Wilcoxon rank-sum statistical test to examine the null hypothesis that the distributions of the two PCs between the individual, harmonized cohort data and the integrated cohort data are common. In all cases, the p-values were larger than 0.05 which denotes that the distributions of the PCs between the individual harmonized cohort data and the integrated cohort data are not significantly different.

### D. Distributed Lymphoma Prediction

To deal with the high imbalance between the lymphoma and non-lymphoma groups (Supplementary Table I), the number of controls was set as twice the number of lymphoma cases per training cohort by downsampling the majority class so that a 1:2 ratio, with age and sex matched controls, is maintained between the case group and the control group (Supplementary Table IV). Due to the small population and in order to take advantage of the statistical power of the whole population on each cohort, the incremental learning process was repeated 5 times using different subsets of controls for the training process each time (Supplementary Table IV).

The incremental learning workflow (Section V) was applied on the harmonized cohort data to develop a distributed lymphoma prediction model using three cohorts for training and one cohort for testing. In order to make the analysis complete, each cohort was included in the testing process by repeating the process four times. For each combination, the XGBoost, Support Vector Machines, Logistic regression, Multinomial Naïve Bayes and Multi-layer Perceptron algorithms were applied in an incremental manner. Then, the optimal combination, i.e., the one with the highest performance in all five algorithms was selected for demonstration purposes, according to which the AOUD, UoA, and UNIPI cohorts were used for training and the HUA cohort for testing. The results are depicted in Supplementary Table V. The lymphoma presence was set as the target to solve a binary classification problem (0: no, 1: yes).

**Fig. 3.**
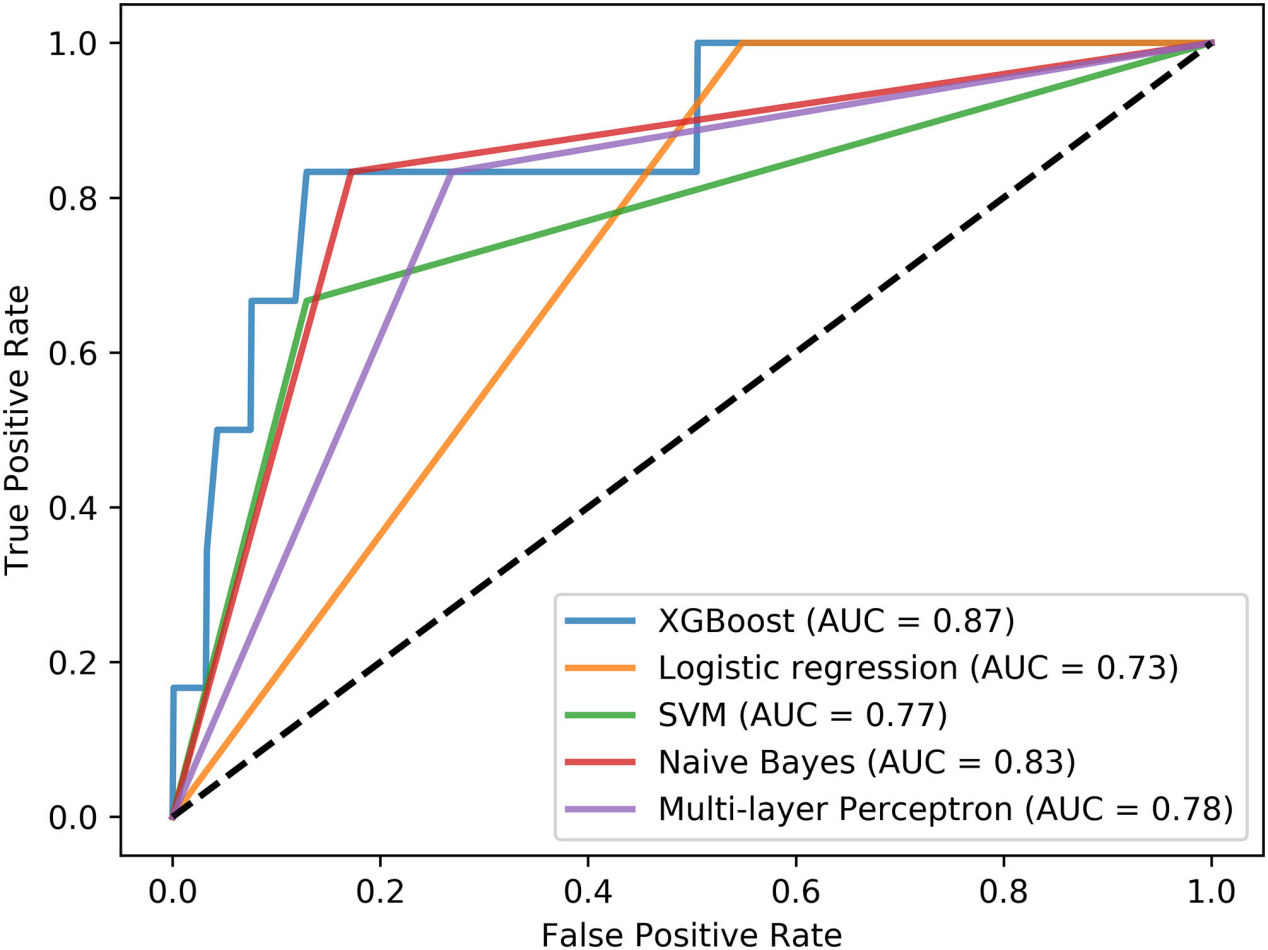
Receiver operating characteristic (ROC) curves for each incremental learning algorithm based on the same training-testing setting.

The performance evaluation measures include the accuracy, sensitivity, specificity and area under the curve (AUC) scores, which are depicted in Supplementary Table V, averaged across 5 runs. According to Supplementary Table V, the XGBoost algorithm (AUC = 0.871, accuracy = 0.859, sensitivity = 0.833, specificity = 0.86) outperforms the rest of the incremental learning algorithms. The Multinomial Naïve Bayes has the second best performance along with the Multi-layer Perceptron. The performance of the Logistic regression and the Support Vector Machines was significantly low in terms of sensitivity. The ROC curves per incremental learning algorithm are depicted in Fig. 3, which confirm the superiority of the XGBoost algorithm.

**Fig. 4.**
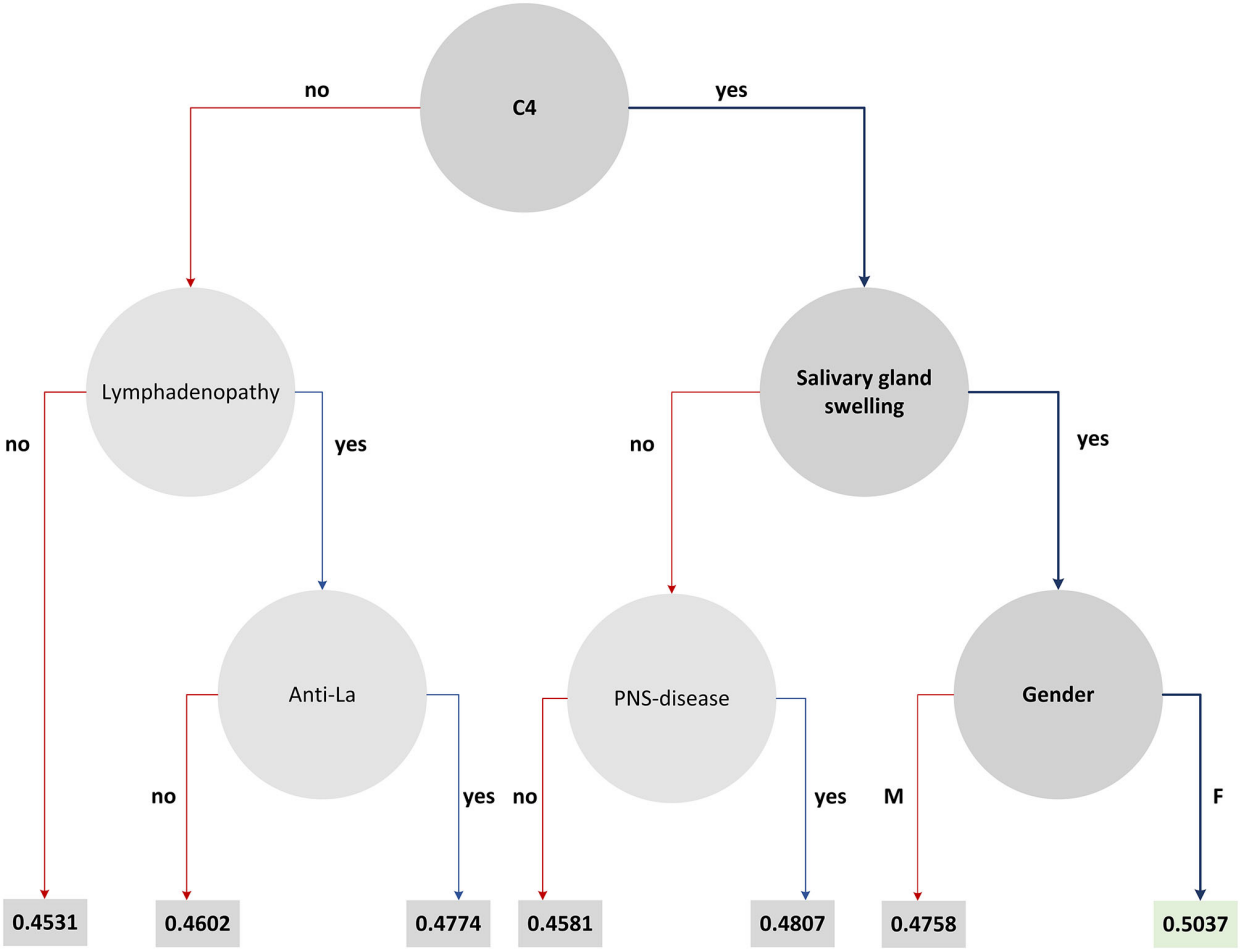
The decision tree that is induced by the XGBoost schema.

To further enhance the clinical findings of the case study we have induced the decision tree from the XGBoost schema which includes the features that highly participated in the decision-making process (Fig. 4). The features with the highest contribution across the splits are represented by a node along with the decision rules, and the rule outcomes (i.e., “yes/no”) are depicted as branches. At the first level lies the “C4” as the root node. The features “lymphadenopathy” and “salivary gland swelling” come next along with the “Anti-La”, and “gender”. The leaf values on each branch denote the conditional probability of a data point falling in class 1 on that branch.

## III. Discussion

**Fig. 5.**
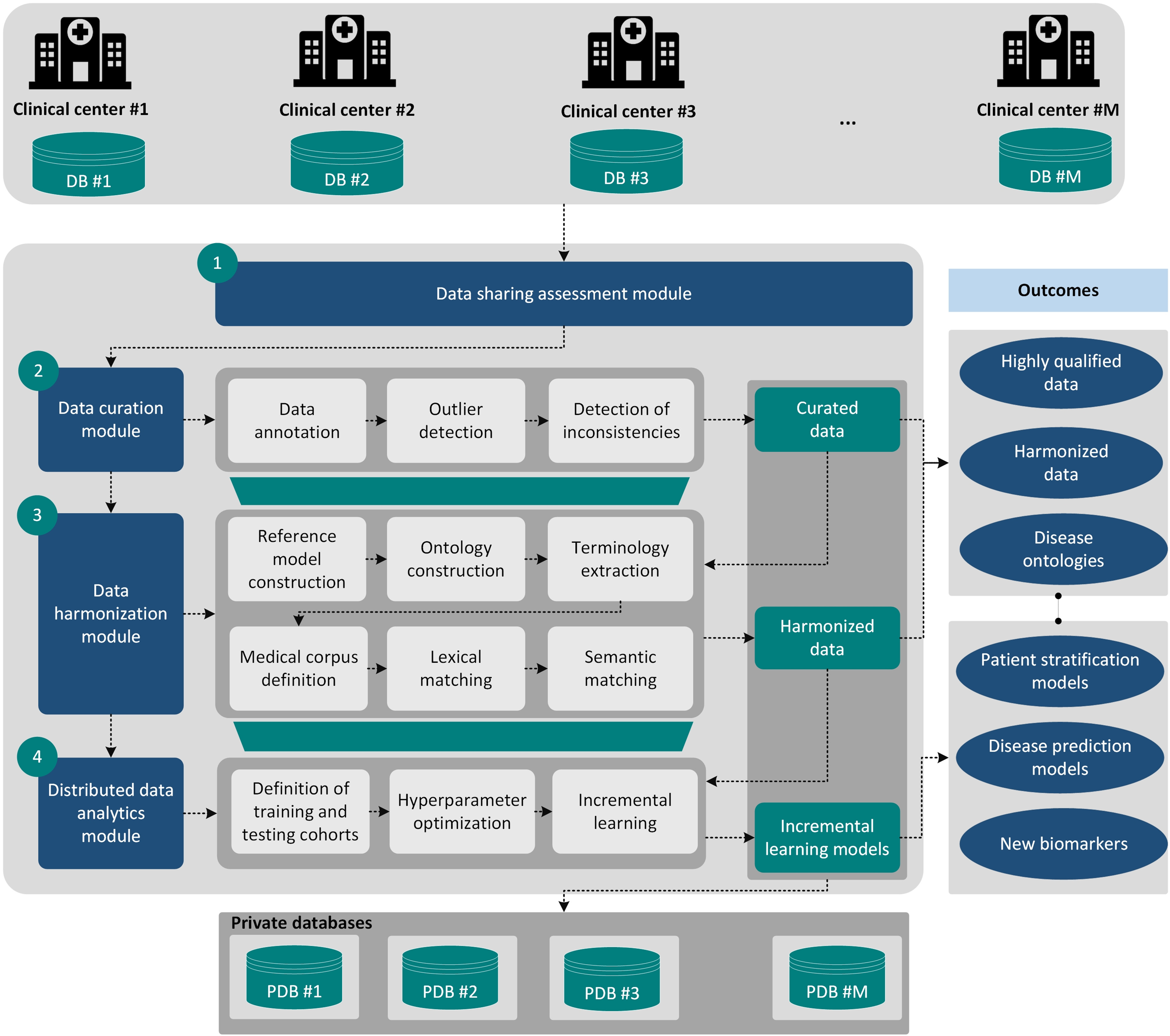
The overall analysis workflow which consists of the following modules: (i) data sharing assessment module, (ii) data curation module, (iii) data harmonization module, and (iv) distributed data analytics module. The workflow receives as input cohort data which are stored in secure private databases within a cloud environment. The outcomes include highly-qualified data, harmonized data, disease ontologies, disease prediction modes, etc.

In this work, we present a computational framework that deals with: (i) the establishment of a GDPR compliant data sharing process, (ii) the enhancement of the quality of the clinical cohort data, (iii) the harmonization of the structurally heterogeneous curated cohort data, and (iv) the application of distributed data analytics workflows on the harmonized cohort data which are stored in private cloud spaces. The applicability of the framework was evaluated on a set of four European cohorts yielding highly-qualified and harmonized cohort data with more than 85% agreement between them along with the development of a lymphoma prediction model having more than 85% accuracy, sensitivity and specificity towards the precise identification of lymphoma cases across highly imbalanced cohort data on pSS. Detailed information regarding the methodology that was used for cohort data harmonization and distributed lymphoma prediction, as part of the data harmonization and distributed data analytics modules (Fig. 5), respectively, can be found in the Supplementary Material section. The latter includes technical information regarding the: (i) reference model construction, (ii) ontology construction, (iii) lexical matching, (iv) semantic matching, and (v) incremental learning followed by related pseudocodes and results.

The establishment of the DCC ensures the legal and ethical compliance of the shared medical data through the in-depth exploitation of the data protection impact assessment documents and the signed consent forms, among others. The value of the data curation workflow was presented in a previous study [12] towards the enhancement of the quality of the clinical data in an automated manner, where its overall impact was compared with existing frameworks in the field.

The data harmonization strategy is semi-automated and requires a reference model as input. This strategy is in line with the majority of the state-of-the-art data harmonization tools, such as, the BiobankConnect software [18], the SORTA [17] and the DataSHaPER [16] which require the definition of a common set of parameters for the domain of interest prior to the harmonization process. The consistency of the harmonized cohort data was demonstrated by the qualitative and quantitative comparison of the principal components between each harmonized cohort dataset and the integrated harmonized dataset, which suggest an increased homogeneity among the harmonized cohort data. The importance of the proposed approach, however, lies on the fact that it is more clinical-oriented and it is based on the definition of a disease-oriented ontology for the purposes of pSS which is a rare, autoimmune disease with a reported lack of domain knowledge instead of the genome-oriented tools, like the DataSHaPER [16] and the BiobankConnect software [18] which are used for the harmonization of genomic data.

An incremental learning strategy is also presented, as part of the distributed data analytics module, which is able to sequentially build machine learning models across harmonized cohort data that are stored in private cloud spaces without the need to construct a centralized repository and thus overcomes data security issues and high-performance computing requirements. The value of the incremental learning strategy is demonstrated in the pSS domain, where there exists a significant lack of lymphoma prediction models due to the rareness of the disease, as well as, due to the increased imbalance between the lymphoma and non-lymphoma pSS patients, a fact that hampers the development of robust lymphoma prediction models for effective disease management. In this work, we extend the incremental learning process from a previous study [36] in a more realistic scenario, where three different harmonized cohort datasets (498 patients) have been recruited to conduct the incremental training process using hyper parameter optimization and a single harmonized cohort dataset was used for testing purposes (99 patients). The XGBoost incremental learning schema achieved the highest accuracy, sensitivity, specificity, AUC scores (Supplementary Table V, Fig. 1), where the Multinomial Naïve Bayes and the Multi-layer Perceptron neural network come next.

According to Supplementary Table V, the logistic regression, Support Vector Machines, and Multilayer Perceptron are less robust due to the escalations that occur around the minimum values a fact that introduces large variations in the loss function and thus in the sensitivity of the final model. This issue, however, is not present in the case of the Multinomial Naïve Bayes, which uses the maximum a-posteriori (MAP) rule for solving the cost minimization problem, as well as, in the XGBoost trees, which deploy tree ensembles to solve a cost function with a regularization parameter that avoids overfitting.

In addition, the fact that the number of lymphomas in the HUA cohort is limited affects mainly the training procedure, because of the large class imbalance that is introduced. Besides, in order to deal with this large class imbalance (caused not only by HUA, but also all cohorts), random undersampling of the majority class was performed, in a ratio 1:2 with age and sex matched controls. Thus, due to the small number of lymphoma cases in that cohort, the ROC curves are steeper.

The clinical outcomes are in line with previous findings [29], [30] which confirm the superiority of the XGBoost in terms of sensitivity and specificity along with the importance of the C4, lymphadenopathy, and rheumatoid factor towards lymphoma prediction. Within the proposed framework the user is also able to query specific subsets of data (e.g., only males, or patients more than 50 years old, or any other type of query) and develop models through training and testing with a specific subset.

## IV. Conclusions

The proposed computational framework provides data quality, data harmonization and data analytics workflows that do not only enhance the statistical power of the heterogeneous clinical data through data curation and harmonization but also applies incremental learning to co-analyze the harmonized data towards the development of robust prediction models for the disease of interest. The framework can be generalized to any clinical domain, as long as, a reference ontology is provided as input towards the construction of a medical corpus which will in turn enable the application of lexical and semantic matching algorithms. As a future work, we are planning to include more harmonized cohort data on the private cloud spaces to further enhance the statistical power of the lymphoma prediction models in the pSS domain, as well as, interlink the corpus with other medical index repositories and include deep learning algorithms in the distributed data analytics module to enhance the robustness of the disease prediction models.

## V. Materials and Methods

### A. The Proposed Analysis Workflow

The overall analysis workflow is depicted in Fig. 5. The cohort data are shared with the framework fulfilling all the necessary legal and ethical requirements which are posed by the GDPR including data protection impact assessment documents and signed consent forms, among others, through a data protection by design (data de-identification) and by default (data minimization) process. The quality of the cohort data is automatically evaluated through the data curation module to annotate the terms and deal with outliers, missing values and inconsistent fields. The curated cohort data are then stored in secure private cloud spaces and then the data harmonization module is executed.

The latter uses lexical and semantic matching algorithms to align lexically and conceptually similar terms between each cohort dataset and a disease-oriented ontology and consists of a six-step sequential process, including: (i) the construction of a reference model, (ii) the transformation of the reference model into a disease-oriented ontology, (iii) the extraction of terminologies from the disease-oriented ontology, (iv) the definition of a medical corpus, and (v) the application of lexical and semantic matching to align the terminologies between the input dataset and the corpus.

Finally, federated data analytics methods are applied for the analysis of the harmonized cohort data in an incremental manner to deal with the unmet needs in the clinical domain of interest through the application of incremental learning algorithms which include additional functionalities for hyper parameter optimization and class imbalance handling. The outcomes of the framework include high quality clinical data, disease-oriented ontologies, harmonized clinical data, machine learning models for patient stratification and disease prediction.

### B. Data Sharing Assessment Module

The data sharing assessment module is responsible for the evaluation of the regulatory compliance of the clinical data with the requirements which are posed by the GDPR for data sharing and data protection [23]. Towards this direction, a data protection by design and by default architecture was adopted along with the set-up of a Data Controllers Committee (DCC) for the examination of the GDPR-related documents including data protection impact assessment documents and signed consent forms, among others.

### C. Data Curation Module

A medical data curation workflow, which was already described in a previous study [12], was applied to deal with outliers, incompatible fields, duplicated fields and missing values within the clinical data.

### D. Data Harmonization Module

**Fig. 6.**
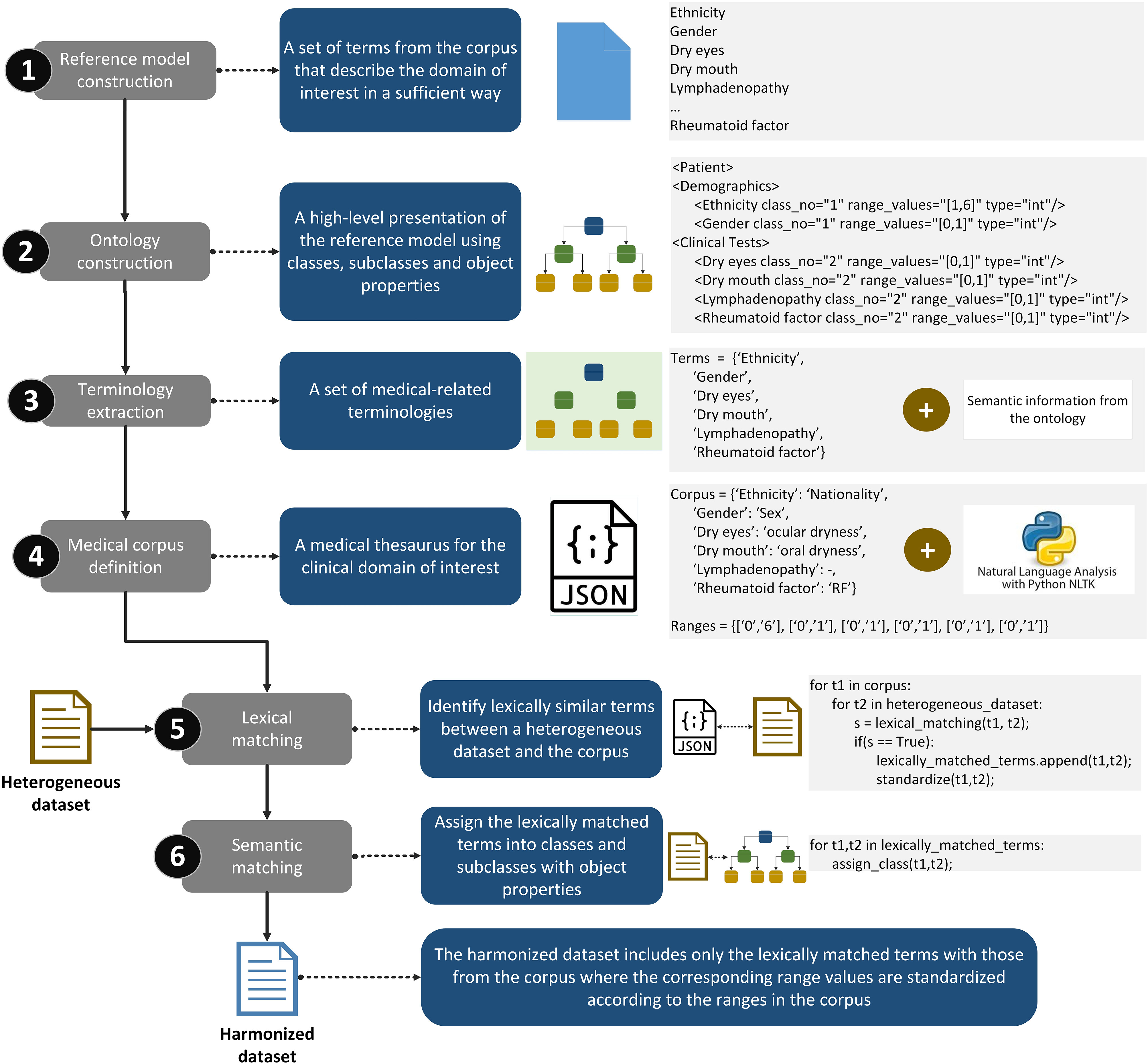
The workflow of the data harmonization module which consists of the following steps: (i) reference model construction, (ii) ontology construction, (iii) terminology extraction, (iv) medical corpus definition, (v) lexical matching, and (vi) semantic matching.

Data harmonization depends on the disease under discussion in this work which is the primary Sjögren's Syndrome (pSS). The proposed strategy, however, can be extended to other medical domains as long as a reference model is available. The data harmonization process is a six-stage process (Fig. 6, Supplementary materials - Section A), where: (i) a reference model is constructed for the medical domain including the parameters that sufficiently describe the domain knowledge [31], [32], (ii) the reference model is transformed into an ontology including classes, subclasses and object properties that hierarchically represent the relation among the parameters, (iii) the terminologies and the range values are extracted from the ontology along with useful semantic information (e.g., class hierarchy), (iv) the terms are used to define a medical corpus (.JSON format) which is enriched with external vocabularies from the Natural Language Toolkit [33], and then given a heterogeneous dataset, (v) lexical matching is applied to match lexically similar terms [34]–[36], and (vi) semantic matching is applied to classify these terms. The outcome is a harmonized dataset which includes the lexically matched variables that are standardized according to the pre-defined range values in the medical corpus.

### E. Distributed Data Analytics Module

**Fig. 7.**
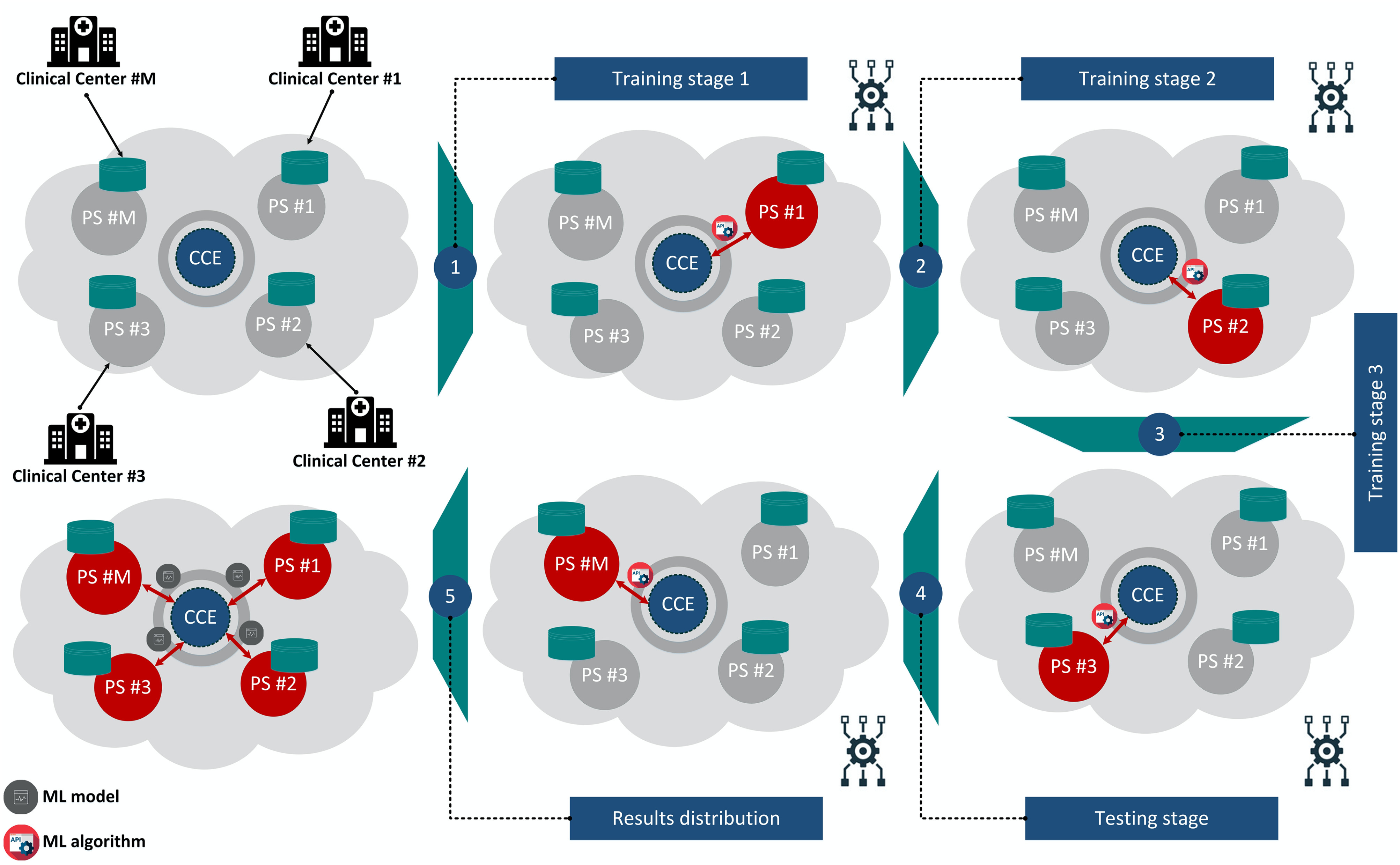
An illustration of the incremental learning strategy within a cloud environment. The data are uploaded into private cloud spaces. The incremental learning model is incrementally updated across the training cohort data through the CCE and is finally evaluated on the testing cohort. The results are distributed to all the cohort that participated in the training-testing setting. CCE: Central Computing Engine, PS: Private Space.

Once the data harmonization process is completed, the harmonized cohort data are stored in secure private cloud spaces [37]. As we already mentioned in the Introduction, we propose an incremental learning strategy to sequentially analyze the harmonized cohort data. According to Fig. 7, a central computing engine (CCE) is used to orchestrate the incremental learning process [36]. The machine learning (ML) model is first trained on the harmonized data that lie in the private space (PS) #1, and then it is incrementally updated across the harmonized data that lie in PS #2, and PS #3, yielding the final model which is evaluated on the data that lie in the PS #M. The ML model is finally distributed to the PSs that participated in process. A pseudocode that summarizes the methodology along with the mathematical background [38]–[42] of the incremental learning algorithms are stated in Supplementary Materials - Section B.

## Supplementary Materials

The Supplementary Materials include technical information regarding the: (i) reference model construction, (ii) ontology construction, (iii) lexical matching, (iv) semantic matching, and (v) incremental learning which are part of the cohort data harmonization and distributed data analytics modules followed by related pseudocodes and results.


