# Supplementary material for: Overcoming the Barriers That Obscure the Interlinking and Analysis of Clinical Data Through Harmonization and Incremental Learning
Source: IEEE Open J Eng Med Biol. 2020 Mar 16;1:83–90. doi: 10.1109/OJEMB.2020.2981258 (PMC8940202; doi:10.1109/OJEMB.2020.2981258)
Supplement: Supplementary file 1 [file supp1-2981258.pdf]

Supplementary Materials

Overcoming the barriers that obscure the interlinking and analysis of clinical data through harmonization and incremental learning

Vasileios C. Pezoulas, *Student Member IEEE*, Konstantina D. Kourou, Fanis Kalatzis, Themis P. Exarchos, *Member IEEE*, Evi Zampeli, Saviana Gandolfo, Andreas Goules, Chiara Baldini, Fotini Skopouli, Salvatore De Vita, Athanasios G. Tzioufas, and Dimitrios I. Fotiadis, *Fellow, IEEE*

A. *Data harmonization module*

1) *Reference model construction*

The reference model is the most common way to describe the domain knowledge of the disease of interest. It is usually defined by the clinical experts as a set of parameters which are able to describe the domain knowledge, in a sufficient way, and thus serves as a gold standard model to enable data harmonization [1-4]. The reference model is a template which consists of medical-related parameters including demographics (e.g., age, gender, ethnicity), laboratory tests (e.g., ocular tests, oral tests, blood tests), conditions (e.g., rheumatoid factor, lymphadenopathy), symptoms (e.g., fever, weight loss), biopsy tests, and interventions (therapies), among many others.

2) *Ontology construction*

The parameters in the reference model are then organized into a semantic, hierarchical way using classes, subclasses and object properties and thus can then be expressed in the form of an ontology to enable semantic matching [1-4]. An ontology is a high-level hierarchical data model, where the parameters in the dataset are organized in a hierarchical manner through classes and sub-classes and the relationship between them is defined by object properties (e.g., a “Patient” “has” “laboratory tests”). The reference ontology can then be expressed in a .XML (eXtensible Markup Language) format [5], which serves as a semantic data model.

3) *Terminology extraction*

The terms from the reference ontology were extracted to define a medical corpus. Semantic information is also extracted including information regarding the class hierarchy and the object properties between the parameters of the ontology.

4) *Medical corpus definition*

The extracted terms are used to define a medical corpus for the medical domain of interest. The medical corpus is enriched with homonymous and synonymous terminologies from the Natural Language Processing Toolkit (NLTK) [6] along with the definition of acronyms for popular laboratory tests (e.g., the acronym “HGB” for the blood test-related parameter “hemoglobin”). In addition, pre-defined range values are also defined for each term to enable data standardization (e.g., “1” for the presence of a condition and “0” otherwise).

5) *Lexical matching*

String similarity scores are computed to detect lexically similar terms between those from the medical corpus and those in the heterogeneous dataset. For a given term, say x, in the heterogeneous dataset, the string similarity is computed between x and every term in the corpus. Here, we use three string similarity metrics, namely the Jaro [7], the Jaro-Winkler [8], and the Levenhstein [9] distance scores.

For two given strings, say a and b, the Jaro string similarity measure [7], $J_{a,b}$, is equal to:

$$J_{a,b} = \begin{cases} 0 & , \quad c = 0 \\ \frac{1}{3} \cdot \left(\frac{x}{|a|} + \frac{x}{|b|} + \frac{x - t}{x} \right), & o/w \end{cases} , \qquad (1)$$

where x is the number of matching (coincident) characters, and t is half the number of transpositions.

The Jaro-Winkler distance measure [8] is a modification of the Jaro distance measure that uses an additional prefix scale c to give more weight to strings with common prefix of a specific length. For two given strings, say a and b, the Jaro-Winkler string similarity measure [8], $JW_{a,b}$, is defined as in:

$$JW_{a,b} = J_{a,b} + (lx(1 - J_{a,b})) , \qquad (2)$$

where $J_{a,b}$ is the Jaro distance, l is the length of common prefix at the start of the string up to a maximum of four characters. The prefix weight is the inverse of the l that is needed to consider both strings as identical.

The Levenhstein distance [9] measures the similarity between two strings, say a and b, in terms of the number of deletions, insertions, or substitutions needed to transform one string to another:

$$L_{a,b}(i,j) = \begin{cases} max(i,j) & , \quad min(i,j) = 0 \\ min \begin{cases} L_{a,b}(i-1,j) + 1 \\ L_{a,b}(i,,j-1) + 1 \\ L_{a,b}(i-1,j-1) + 1_{(a_i \neq b_j)} \end{cases} & , \quad o.w. \end{cases} . \qquad (3)$$

Here, we use an empirical combination of these three string similarity scores to take advantage of each method’s individual properties towards the robust detection of lexically identical or similar terms having: (i) Jaro distance larger than (or equal to) 0.8 and Levenhstein distance less than 2 or (ii) Jaro distance larger than 0.9 or (iii) Jaro-Winkler distance 1 and Jaro distance larger than 0.85. Of course, in the case where the terms have a Jaro distance 1 they are considered as exactly matched. The

thresholds have been derived after numerous experimentations towards the identification of the maximum number of lexically identical terms. Since the Jaro-Winkler distance score is more strict due to the fact that it gives more weight to the terms with common continuous characters we used a smaller threshold than the one in the Jaro distance score.

6) Semantic matching

Semantic matching is the process of matching terms which share a common conceptual basis. Each term in the reference model is followed by a class ID which describes the term's concept through a "consist of" property whereas an "includes" property denotes the interlinked class. For example, a "Laboratory Test" "consists of" a "Blood test" which "includes" the variable "hemoglobin". A pseudocode for harmonization is presented in Algorithm 1. The pseudocode requires as input the terms and values of the heterogeneous dataset along with the reference ontology. The object properties and classes are first extracted from the ontologies and the variables are extracted by the object property "includes" and "consists of", respectively. The variables are enriched with synonymous terms from the NLTK database along with acronyms from the clinical experts. Lexical matching is then applied to seek for lexical similarities between the medical corpus and the variables from the given dataset. Once a match is found, the common terms are stored into a list along with the indices and standardization is applied to compute the new values. The harmonized data are finally written into a new file given the matched variables, the matched indices and the new values.

Supplementary Algorithm 1. A pseudocode for lexical and semantic matching.

```
def harmonization(terms, values, onto):
    prop = onto.object_properties;
    vars = [x[i] for i, x in prop if x == "includes"];
    class = [x[i] for i, x in prop if x == "consists of"];
    syns = [nltk.synsets[vars[i]] for i, x in vars];
    med_corpus = {vars U syns};
    for i in len(vars):
        for j in len(med_corpus):
            if (lexical_match(vars[i], med_corpus[j])):
                m_vars[k] = list([vars[i], med_corpus[j]]);
                m_classes[k] = list([vars[i], class[j]]);
                stand_values[k] = standardize(vars[i],
                                  med_corpus[j], values[j]);
    write_data(m_vars, m_classes, stand_values);
end
```

B. Distributed data analytics module

1) Definition of the training and testing cohorts

According to the definition of incremental learning, its mathematical basis can be extended towards the development of machine learning models across data that are stored in different locations. Given a set of M-databases, say $\boldsymbol{D_1}, \boldsymbol{D_2}, \dots, \boldsymbol{D_M}$, with datasets, say $\boldsymbol{x_1}, \boldsymbol{x_2}, \dots, \boldsymbol{x_M}$, a machine learning model, say $\boldsymbol{M_1}$, is initially trained on the dataset $\boldsymbol{x_1}$ in database $\boldsymbol{L_1}$ and then update the model through the update function:

$$f(j) = f(j-1) + ah(j), \quad (4)$$

where $f(j)$ corresponds to the weighted cost function on the dataset in site $\boldsymbol{L_j}$, $f(j-1)$ corresponds to the estimated weighted cost function which was trained on the dataset in the previous site $\boldsymbol{L_{j-1}}$ and $h(j)$ is the learner function on the dataset in database $\boldsymbol{L_j}$. The majority of the supervised learning algorithms use the stochastic gradient descent to minimize a loss function by solving the following weight update process:

$$w(j) = w(j-1) - \alpha(\nabla_w L(f(x_i), y_i) + \beta \nabla_w r(w)), \quad (5)$$

where $L(f(x_i), y_i)$ is a loss function given a score function $f(x_i)$ and a target y_i, $\nabla_w L(f(x_i), y_i)$ is the gradient of the loss function with respect to w, $r(w)$ is a regularization function, $\nabla_w r(w)$ is the gradient of the regularization function with respect to w, β is a hyperparameter, and α is a learning rate parameter.

A pseudocode that summarizes the overall methodology is presented in Algorithm 2. A machine learning algorithm in the form of an object, M_0, is initialized and trained on the dataset X_1 in PS #1. Then, the machine learning model is updated on each site according to (4) and (5) and the final model is stored to each PS that participated in the incremental learning process.

Supplementary Algorithm 2. A pseudocode for the incremental learning process.

initialize an ML algorithm object, say $\boldsymbol{M_0}$;
train $\boldsymbol{M_0}$ on dataset $\boldsymbol{X_1}$ in PS #1 yielding the model $\boldsymbol{M_1}$;
for i = 1, 2, …, M-1:
    establish a secure connection with the i-th PS;
    compute the updated weights according to (4);
    update $\boldsymbol{M_i}$ on dataset $\boldsymbol{X_{i+1}}$ in the $i+1$-th PS yielding model $\boldsymbol{M_{i+1}}$ using (5);
    store the model $\boldsymbol{M_{i+1}}$ for updating on the $i+1$-th PS;
return $\boldsymbol{M_{M-1}}$;

According to the optimal schema, each machine learning algorithm was initially trained on the harmonized data from the AOUD cohort (with hyper parameter optimization) yielding the ML model #1. The latter model was updated on the harmonized data from the UoA cohort yielding the ML model #2 which was finally updated on the harmonized data from the UNIPI cohort yielding the ML model #3. The final model was applied on the harmonized cohort data from the HUA cohort for performance evaluation,

2) Hyper parameter optimization

Hyper parameter optimization was applied on the first training cohort dataset to fine tune the incremental learning algorithms. More specifically, the randomized search on hyper parameters method [10] was applied, where the number of parameter settings was set to 100. A 10-fold cross-validation strategy was then applied to evaluate the parameter settings on the harmonized data. For example, in the logistic regression and the Support Vector Machines classifiers, the elastic net, l1 and l2 penalties were evaluated along with the maximum iteration number towards convergence, the alpha values, the learning

rates and the tolerance of each classifier, as described in [11]. For the Multi-layer Perceptron classifier the additional parameters that were evaluated include the activation function and the solver function [11].

3) *Incremental learning algorithms*

a) *Stochastic gradient descent*

Given a set of training observations, $\{(\boldsymbol{x_1},y_1), (\boldsymbol{x_2},y_2), \dots, (\boldsymbol{x_M},y_M)\}$, where $\boldsymbol{x_i} \in \boldsymbol{R^M}$, and an outcome y_j, where $y_j \in \{-1,+1\}$, the goal of the stochastic gradient descent (SGD) approach is to seek for a linear loss function, say $\varphi\left(f(\boldsymbol{x_j}), y_j\right)$, that minimizes the equation [12]:

$$\Phi(\boldsymbol{w}) = argmin\left(\frac{1}{M}\sum_{j=1}^{M}\varphi\left(f(\boldsymbol{x_j}), y_j\right) + ar(\boldsymbol{w})\right), \qquad (6)$$

where $\boldsymbol{w}$ is a weight vector, $\varphi(.)$ is a loss function, a is a non-negative hyperparameter, $r(\boldsymbol{w})$ is a regularizer, and $f(\boldsymbol{x_j})$ is a linear score function in the form $\boldsymbol{w}^T\boldsymbol{x_j}$.

Solving (6) for the weight vector we get the SGD weight update rule [12]:

$$\widetilde{\boldsymbol{w}}_{\boldsymbol{i}} = \widetilde{\boldsymbol{w}}_{\boldsymbol{i-1}} - \eta_t\left(\frac{\partial\varphi\left(f(\boldsymbol{x_j}), y_j\right)}{\partial\boldsymbol{w}} + a\frac{\partial r(\boldsymbol{w})}{\partial\boldsymbol{w}}\right), \qquad (7)$$

where i is the time step, $\widetilde{w}_{i-1}$ is the weight estimation at step $i-1$, η_i is a non-negative learning rate parameter, and $\varphi'(.)$ is the gradient of the loss function $\varphi(.)$. The cost function, $\varphi\left(f(\boldsymbol{x_j}), y_j\right)$, in (7) can be replaced by various cost functions to obtain a specific classifier. For example, if we set:

- $\varphi\left(f(\boldsymbol{x_j}), y_j\right) = ln\left(1 + exp\left(-y_j f(\boldsymbol{x_j})\right)\right)$, we can obtain the logistic regression classifier.
- $\varphi\left(f(\boldsymbol{x_j}), y_j\right) = max\left(0, 1 - y_j f(\boldsymbol{x_j})\right)$, we can obtain the Support Vector Machines classifier.
- $\varphi\left(f(\boldsymbol{x_j}), y_j\right) = max\left(0, -y_j f(\boldsymbol{x_j})\right)$, we can obtain the Perceptron classifier.

b) *Multinomial Naïve Bayes*

Given an N-dimensional input vector, assume $\boldsymbol{f} = (f_1, f_2, \dots, f_N)$, where each f_j is the frequency of an event $\boldsymbol{x_j}$, and the probability that $\boldsymbol{f}$ belongs to the class, assume c_k, is the multinomial distribution, $P(c_k|\boldsymbol{f})$, we can calculate the class with the highest probability or the maximum a-posterior (MAP) class, as in [13]:

$$c_{MAP} = argmax_{c_k}\left[log\left(P(c_k)\right) + \sum_{j=1}^{N} log\left(P(\boldsymbol{x_j}|c_k)\right)\right], \qquad (8)$$

where $P(\boldsymbol{x_j}|c_k)$ is the conditional probability of x_j occurring in class c_k. It is obvious that (8) can be solved as a linear equation [13].

c) *Gradient Boosting Trees*

The Gradient Boosting algorithm [14, 15] is an ensemble classifier which combines a set of weak learners into a stronger classifier where on each boosting round the algorithm minimizes the gradient of a loss function to minimize the prediction errors and thus optimize the overall performance of the classifier. Gradient Boosting adopts a regularized approach to further reduce the over-fitting on the gradient and thus enhance the overall performance of the classifier. The gradient boosting classifier seeks for a weak learner, at step i, say $f_i(x)$, so that:

$$F_i(x) = F_{i-1}(x) + argmin_f\left(\sum_{j=1}^{n} L\left(\tilde{y}_j, F_{i-1}(x_j) + f_i(x_j)\right)\right), \qquad (9)$$

where $L(y, F(x))$ is the error loss function and n is the number of samples and $\tilde{y}_j$ is the estimated target value for the j-th sample. Eq. (9) can be re-written as:

$$F_i(x) = F_{i-1}(x) + \gamma_i f_i(x), \qquad (10)$$

where $F_{i-1}(x)$ is the weak learner at step $i-1$ and γ_i is calculated by minimizing the gradient descent:

$$\gamma_i = argmin_\gamma\left(\sum_{j=1}^{n} L\left(y_j, F_{i-1}(x_j) - \gamma\nabla_{F_{i-1}}L\left(y_j, F_{i-1}(x_j)\right)\right)\right). \qquad (11)$$

Substituting (11) into (10) yields the following learner:

$$F_i(x) = F_{i-1}(x) - \gamma_i\sum_{j=1}^{n}\nabla_{F_{i-1}}L\left(y_j, F_{i-1}(x_j)\right). \qquad (12)$$

The Gradient Boosting algorithm is often appeared using a classification tree as a weak learner [39, 40]:

$$F_i(\boldsymbol{x}) = F_{i-1}(\boldsymbol{x}) + \gamma_i\sum_{l=1}^{L} b_{li}\boldsymbol{1}(\boldsymbol{x} \in R_{li}), \qquad (13)$$

where at step i, a regression tree partitions the original space into L-disjoint regions $\{R_{lm}\}_1^L$, where R_{lm} is the L-terminal node, b_{li} is the value predicted in each region R_{lm}, and $\boldsymbol{1}(.)$ is an indicator function (1:true, 0:otherwise). In this work, the XGBoost library [15] was used to incrementally train a gradient boosting tree across the harmonized data along with Python's scikit library [16] which was used for the incremental application of the rest of the algorithms. The incremental application of the XGBoost algorithm offers a robust ensemble approach that iteratively builds decision trees over the harmonized data with reduced prediction errors based on the gradient direction (gradient boosting).

C. *Supplementary tables*

Supplementary Table I. Demographic information.

	UoA	HUA	UNIPI	AOUD
**Age at diagnosis**	53.5±13.5	47.5±12.3	51.4±14	52.4±13.9
**Gender ratio (females/ males)**	415/25	96/3	693/25	274/23
**Lymphoma/ Non-lymphoma (%)**	76/364 (20.87%)	6/93 (6.45%)	31/687 (4.51%)	26/271 (9.59%)
**Total number of patients**	440	99	718	297

Supplementary Table II. Extracted cohort metadata.

	UoA	HUA	UNIPI	AOUD
**Number of features**	167	204	102	82

**Number of instances (cases)**	440	100	718	297
**Categorical features**	76	146	85	75
**Numeric features**	60	52	17	6
**Good features**	27	62	37	51
**Fair features**	59	74	50	17
**Bad features***	81	68	15	14
**Features with outliers**	0	0	0	0
**Features with inconsistencies**	31	6	0	1
**Total % of missing values**	44.8%	33.61%	21.98%	17.15%

*these features were discarded from further analysis.

Supplementary Table III. Cohort data harmonization results.

	Cohorts			
	**UoA**	**HUA**	**UNIPI**	**AOUD**
**Number of terms ***	82	136	87	67
**Number of relevant terms with the pSS reference model ****	39	42	54	46
**Number of lexically similar terms with those from the ontology**	36	38	48	41
**Percentage of harmonized terms**	92.3%	90.47%	88.88%	89.13%
**Number of terms requiring data standardization *****	2	3	1	14
**Common number of terms ********	19			

* after the removal of terms having more than 50% missing values (through data curation).
** the number of pSS-relevant terms for each cohort was identified by the clinical experts (after evaluation).
*** the number of terms that require data transformation according to the range values in the ontology.
**** the number of common harmonized terms (not individual terms) across the cohorts.

Supplementary Table IV. Overall population characteristics for distributed lymphoma prediction.

	Cohorts			
	Set of training cohorts			Testing cohort
	**AOUD**	**UoA**	**UNIPI**	**HUA**
**Number of lymphoma cases**	26	76	31	6
**Number of controls ***	52	152	62	93
**Total population**	78	228	93	99

* the number of controls was randomly selected 5 times to "cover" each cohort's population during training.

Supplementary Table V. Performance evaluation scores per incremental learning algorithm and testing cohort combination.

Algorithm	Testing cohort	Accuracy	Sensitivity	Specificity	AUC
**XGBoost**	AOUD	0.835	0.692	0.849	0.849
	UoA	0.834	0.526	0.898	0.898
	UNIPI	0.872	0.484	0.889	0.889
	HUA	0.859	0.833	0.86	0.871
**Logistic regression**	AOUD	0.926	0.462	0.97	0.97
	UoA	0.805	0.184	0.934	0.934
	UNIPI	0.85	0.29	0.875	0.875
	HUA	0.899	0.167	0.946	0.556
	AOUD	0.902	0.692	0.923	0.923
	UoA	0.714	0.684	0.72	0.72
**Support Vector Machines**	UNIPI	0.948	0.032	0.99	0.99
	HUA	0.768	0.667	0.774	0.72
**Multinomial Naïve Bayes**	AOUD	0.869	0.846	0.871	0.871
	UoA	0.823	0.5	0.89	0.89
	UNIPI	0.799	0.484	0.814	0.814
	HUA	0.828	0.833	0.828	0.831
**Multilayer Perceptron**	AOUD	0.875	0.808	0.882	0.882
	UoA	0.839	0.263	0.959	0.959
	UNIPI	0.879	0.387	0.901	0.901
	HUA	0.747	0.833	0.742	0.788

References

[1] K. D. Kourou, V. C. Pezoulas, E. I. Georga, T. P. Exarchos, P. Tsanakas, M. Tsiknakis, T. Varvarigou, S. De Vita, A. Tzioufas, and D. I. Fotiadis, "Cohort harmonization and integrative analysis from a biomedical engineering perspective," IEEE reviews in biomedical engineering, vol. 12, pp. 303-318, 2018.

[2] V. C. Pezoulas, T. P. Exarchos, V. Andronikou, T. Varvarigou, A. G. Tzioufas, S. De Vita, and D. I. Fotiadis, "Towards the establishment of a biomedical ontology for the primary Sjögren's Syndrome," In 40th Annual International Conference of the IEEE Engineering in Medicine and Biology Society, pp. 4089-4092, 2018.

[3] I. Fortier, P. Raina, E. R. van den Heuvel, L. E. Griffith, C. Craig, and M. Saliba, "Maelstrom research guidelines for rigorous retrospective data harmonization," International Journal of Epidemiology, vol. 46, pp. 103-105, 2017.

[4] M. Zhao, S. Zhang, W. Li, G. Chen, "Matching biomedical ontologies based on formal concept analysis," Journal of biomedical semantics, vol. 9, no. 1, pp. 11, 2018.

[5] J. Paoli, C. M. Sperberg-McQueen, F. Yergeau, E. Maler, and T. Bray, "Extensible markup language (xml) 1.0," W3C recommendation, 2004.

[6] E. Loper, and S. Bird, "NLTK: the natural language toolkit," 2002. arXiv preprint cs/0205028.

[7] S. J. Gandhi, M. M. Thakor, J. Sheth, H. I. Pandit, and H. S. Patel, "Comparison of String Similarity Algorithms to Measure Lexical Similarity," NJSIT, vol. 10, no. 2, pp. 139, 2017.

[8] M. Cheatham, and P. Hitzler, "String similarity metrics for ontology alignment," In International semantic web conference, pp. 294-309, Springer, Berlin, Heidelberg, 2013.

[9] G. A. Rao, G. Srinivas, K. V. Rao, and P. P. Reddy, "Characteristic Mining of Mathematical Formulas from Document-A Comparative Study on Sequence Matcher and Levenshtein Distance Procedure," International Journal of Computer Sciences and Engineering, vol. 6, no. 4, pp. 400-403, 2018.

[10] J. Bergstra, and Y. Bengio, "Random search for hyper-parameter optimization," Journal of Machine Learning Research, pp. 281-305, 2012.

[11] F. Pedregosa, G. Varoquaux, A. Gramfort, V. Michel, B. Thirion, O. Grisel, and J. Vanderplas, "Scikit-learn: Machine learning in Python," Journal of machine learning research, pp. 2825-2830, 2011.

[12] L. Bottou, "Large-scale machine learning with stochastic gradient descent," In COMPSTAT'2010 Proceedings, Physica-Verlag HD, pp. 177-186, 2010.

[13] C. D. Manning, P. Raghavan, and H. Schütze, "Introduction to information retrieval," Cambridge University Press, Cambridge, 2008.

[14] J. H. Friedman, "Greedy function approximation: a gradient boosting machine," Annals of statistics, vol. 29, pp. 1189-1232, 2001.

[15] T. Chen, T. He, M. Benesty, V. Khotilovich, and Y. Tang, "Xgboost: extreme gradient boosting," R package version 0.4-2, pp. 1-4, 2015.

[16] F. Pedregosa, G. Varoquaux, A. Gramfort, V. Michel, B. Thirion, O. Grisel, and J. Vanderplas, "Scikit-learn: Machine learning in Python," Journal of machine learning research, pp. 2825-2830, 2011.
